# Evaluation of two different transarterial chemoembolization protocols using Lipiodol and degradable starch microspheres in therapy of hepatocellular carcinoma: a prospective trial

**DOI:** 10.1007/s12072-021-10193-8

**Published:** 2021-05-27

**Authors:** T. J. Vogl, M. C. Langenbach, R. Hammerstingl, M. H. Albrecht, A. R. Chatterjee, T. Gruber-Rouh

**Affiliations:** 1 Institute for Diagnostic and Interventional Radiology, Johann Wolfgang Goethe University, Theodor-Stern-Kai, 760590 Frankfurt am Main, Germany; 2 Department of Radiology and Radiological Science, Medical University of South Carolina, Charleston, SC USA

**Keywords:** Hepatocellular carcinoma, TACE, Lipiodol, DSM, Mitomycin C, Magnetic resonance imaging, Response, Survival, Tumor volume, Necrotic area

## Abstract

**Background:**

This prospective randomized trial is designed to compare the performance of conventional transarterial chemoembolization (cTACE) using Lipiodol-only with additional use of degradable starch microspheres (DSM) for hepatocellular carcinoma (HCC) in BCLC-stage-B based on metric tumor response.

**Methods:**

Sixty-one patients (44 men; 17 women; range 44–85) with HCC were evaluated in this IRB-approved HIPPA compliant study. The treatment protocol included three TACE-sessions in 4-week intervals, in all cases with Mitomycin C as a chemotherapeutic agent. Multiparametric magnetic resonance imaging (MRI) was performed prior to the first and 4 weeks after the last TACE. Two treatment groups were determined using a randomization sheet: In 30 patients, TACE was performed using Lipiodol only (group 1). In 31 cases Lipiodol was combined with DSMs (group 2). Response according to tumor volume, diameter, mRECIST criteria, and the development of necrotic areas were analyzed and compared using the Mann–Whitney-U, Kruskal–Wallis-H-test, and Spearman-Rho. Survival data were analyzed using the Kaplan–Meier estimator.

**Results:**

A mean overall tumor volume reduction of 21.45% (± 62.34%) was observed with an average tumor volume reduction of 19.95% in group 1 vs. 22.95% in group 2 (*p* = 0.653). Mean diameter reduction was measured with 6.26% (± 34.75%), for group 1 with 11.86% vs. 4.06% in group 2 (*p* = 0.678). Regarding mRECIST criteria, group 1 versus group 2 showed complete response in 0 versus 3 cases, partial response in 2 versus 7 cases, stable disease in 21 versus 17 cases, and progressive disease in 3 versus 1 cases (*p* = 0.010). Estimated overall survival was in mean 33.4 months (95% CI 25.5–41.4) for cTACE with Lipiosol plus DSM, and 32.5 months (95% CI 26.6–38.4), for cTACE with Lipiodol-only (*p* = 0.844), respectively.

**Conclusions:**

The additional application of DSM during cTACE showed a significant benefit in tumor response according to mRECIST compared to cTACE with Lipiodol-only. No benefit in survival time was observed.

## Introduction

Hepatocellular carcinoma [HCC] is one of the most common malignancies with an annual worldwide incidence of 700,000 new cases [1, 2]. The common pathway for therapy of HCC involves a multidisciplinary tumor board deciding the treatment pathway, curative vs. non-curative, based on patients' status and the Barcelona-Clinical Liver Cancer [BCLC] staging system [3]. Since many patients are diagnosed in an advanced stage of the disease, or with severely reduced hepatic conditions, the prognosis of HCC remains poor. Curative approaches like surgical resection, liver transplantation or ablation, for example using radiofrequency or microwave systems, are reserved for HCC in an early stage. For intermediate stage tumors (BCLC stage B), conventional Lipiodol-based trans-arterial chemoembolization [cTACE] is recommended, either if the tumor is unresectable or as bridge-to-therapy in a potentially curative situation [4]. For advance stage diseases (BCLC stage C), Sorafenib, a multiple tyrosine kinase inhibitor, is recommended for treatment. Patients with an extensive disease in terminal stage (BCLC stage D) should receive best supportive care in a palliative setting.

Currently, there is a clinical equipoise for patients in BCLC stage B regarding the most effective technical steps of the TACE procedure with different techniques performed in an attempt to maximize the tumor response. Various methods of TACE are established worldwide with different embolic and chemotherapeutic agents [5]. In general, all strategies use the occlusion of the tumor-feeding artery immediately following or during intra-arterial administration of the chemotherapeutic agent to minimize the wash-out of therapeutics. Best investigated and widely spread is the transarterial chemoembolization with Lipiodol only. Another notably method is the administration of drug-eluting beats (DEB) for embolization, named DEB-TACE. A third alternative approach is the use of degradable starch microspheres (DSM) for embolization, DSM-TACE. For DEB- and DSM-TACE, microspheres are used for embolization instead of or additional to Lipiodol. However, post-interventional vascular endothelial growth factor [VEGF]-induced tumor arterial collateralization represents a challenge to its effectiveness. With knowledge of the pathophysiological background, temporary occlusion of the tumor-feeding arteries using DSM has been proposed to minimize the VEGF-induced tumor neovascularization with good transient embolization in the post-intervention high effective phase of the chemotherapeutics [6]. Consecutive, an additional application of DSM combined with Lipiodol might connect the benefits of a transient and long-lasting embolization with a reduced tumor neovascularization [7].

Concluding, we hypothesized that in patients undergoing TACE for treatment of HCC, the combined application of Lipiodol and DSM (cTACE plus DSM) would improve treatment response. Thus, the aim of this study was to investigate the effect of cTACE + DSM compared to cTACE with Lipiodol-only by monitoring the local tumor response and survival in patients with HCC. This is a novel and unique approach in the interventional therapy of HCC.

## Patients and methods

### IRB

This prospective randomized clinical HIPPA-compliant study was approved by our local ethical committee. All participants gave written informed consent prior to inclusion, both for treatment with TACE and study inclusion.

### Participant enrollment and randomization

All patients presenting to our institution with HCC for whom TACE was recommended by a multidisciplinary tumor board according to BCLC criteria were screened for inclusion in the study [3]. Also included were participants for whom TACE was performed as bridging treatment according to Milan-criteria [8].

Inclusion criteria were: age > 18 years, radiologically or histologically proven diagnosis of HCC, and tumor size > 1 cm according to an existing MRI scan. Exclusion criteria were: inability to obey breathing commands, any general MRI contraindications such as a cardiac pacemaker or metal implants, a GFR ≤ 30 ml/min, known allergy to contrast media, and a second malignancy.

Participants were randomized into two arms, cTACE with Lipiodol-only (Lipiodol-TACE) and Lipiodol in combination with DSM (cTACE plus DSM). Randomization was performed using a randomization list for each patient eligible for this study. The list was never visible to any investigator of this trial. Enrollment size was estimated with an expected clinical relevant difference of 0.75 standard deviations and a power of 0.80 with consideration of the Bonferroni correction for all primary endpoints. This resulted in 29 patients per group, with consideration of the ARE-correction 31 patients (in total 61 patients). The study was performed as a pilot study.

### TACE-therapy

Following catheter access of the femoral artery with Seldinger technique and angiography of the abdominal arterial vasculature, proceeding through the celiac trunk a catheter was positioned in the common hepatic artery. The catheter was then advanced through the segmental and subsegmental hepatic branches to the most distal possible tumor-accommodating segmental artery in a superselective approach where the chemotherapeutic-Lipiodol-mix plus/minus DSM was injected. In the case of bilateral affected liver lobes, the treatment was performed to control the lobe with the higher tumor burden as determined by the MRI performed immediately before the procedure.

In both groups, a suspension of Mitomycin C (Medac®, Hamburg, Germany) and the embolization agent Lipiodol (Guerbet GmbH, France) was initially injected in the ratio 1:2 [9]. In the cTACE plus DSM group, the DSM-particle EmboCept® S (PharmaCept GmbH, Berlin, Germany) with a microsphere mean size of 50 µm at a volume of 3.0 ml (180 mg) was subsequently injected to devascularize the tumor-accommodative arteries and the tumor edge area. The maximum amount of the chemotherapeutic agent Mitomycin C administered was 8 mg/m^2^ [10]. A maximum of 5 ml Lipiodol was administered per session in both groups. Endpoint of the administration was stasis in the embolized vessel.

After embolization, a further angiography confirmed the devascularization of the tumor feeding arteries. A CT scan to evaluate the hepatic Lipiodol opacification and to monitor side effects such as Lipiodol-carryover was performed 24 h after every TACE.

Repetitive TACE treatments were performed in 4 week intervals. All patients were treated as outpatients. Complications were noted and rated according to the severity score based on SIR latest classification: 0 = no complications, 1 = mild adverse events (no/non-substantial therapy required), 2 = moderate adverse event (substantial treatment required), 3 = severe adverse events (escalation of care), 4 = life-threatening or disabling event, 5 = patient death [11].

### MRI evaluation

The morphologic tumor response was evaluated by MRI on a single Siemens 1.5 T Magnetom Avanto scanner (Erlangen, Germany) using a body coil (Body 18; Siemens, Erlangen, Germany). Initial treatment planning, short-term status evaluations prior to every TACE cycle, and final treatment result MRI protocols were all the same with unenhanced and contrast-enhanced T1 sequences with 0.1 mmol/kg body weight of gadoteric acid (Dotarem®, Guerbet GmbH, Sulzbach, Germany) or gadobutrol (Gadovist®, Bayer Vital GmbH, Leverkusen, Germany). The detailed sequence protocol is given in Table 1.

**Table 1:** Pre- and post-treatment MRI Protocol

Localizer in 3 levels
T2W, axial and coronal
T1W-FLASH-2D, axial
EP-2D-Diffusion (b50, b400, b800)
T1W-3D native, axial
Contrast administration
T1W-3D, 3-phase post-contrast, axial
T1W-FLASH-2D, axial, post-contrast

### End points

The major endpoint of this study was a change of the target lesion following therapy in each of the two groups with a dedicated analysis of tumor volume, diameter, mRECIST-criteria, and intra-tumoral necrotic area.

Secondary endpoints were the evaluation of volume, mRECIST, and necrotic area, within each group.

### MRI analysis

All MRI evaluations including the response evaluation criteria in solid tumors (RECIST) 1.1-criteria and quantitative measurement of necrotic areas were performed by two radiologists with more than 5 and 12 years of experience in abdominal imaging in consensus.

For the evaluation of diameter, volume and necrotic areas, Volume Viewer 2 (AW Suite 2.0, GE, Chalfont, UK) was used. mRECIST is defined as the ratio between the longest contrast-enhancing diameter in the final MRI and in the initial MRI. The progress was analyzed according to the mRECIST protocol [12].

### Statistics

Statistical analysis was performed using SPSS V25.0 (IBM, Armonk, NY, USA). Descriptive statistics include mean and median of volume, diameter, and necrotic areas. Differences between both groups were assessed using a non-parametric Mann–Whitney-U-Test. mRECIST criteria differences were assessed with Kruskal–Wallis-H-Test with post-hoc-analysis. Correlations were evaluated using Spearman-Rho, and the correlation coefficient was classified according to Evans classification [13, 14]. Survival curves were prepared using the Kaplan Maier method. For group comparison, the log-rank Mantel-Cox test was used. *p* < 0.05 was considered significant.

## Results

### Participants

Sixty-two patients (44 men; 18 women; mean age 71 years; range 44–85 years) who met the inclusion criteria with HCC were included. One individual was excluded due to an unclear entity of the lesion based on the morphological appearance in the acquired imaging. All other 61 patients met inclusion criteria, were randomized and analyzed (Table 2).

**Table 2:** Baseline patient characteristics

Characteristic	Lipiodol, N (%)	Lipiodol + DSM, N (%)	Total, N (%)	*p*-value
No. of patients	30	31	61	
Sex				0.838
Male	22 (73.3)	22 (71.0)	44 (72.1)	
Female	8 (26.7)	9 (29.0)	17 (27.9)	
Age (years)				0.795
Mean (range)	72 (44–85)	70.5 (47–85)	71 (44–85)	
Fundamental disease				0.538
Hepatitis B	6 (20)	5 (16.1)	11 (18)	
Hepatitis C	11 (36.7)	11 (35.5)	22 (36.1)	
Ethyltoxic	9 (30)	9 (29)	18 (29.5)	
Hepatitis C + Ethyltoxic	2 (6.7)	2 (6.5)	4 (6.6)	
NASH	2 (6.7)	3 (9.7)	5 (8.2)	
PBC	0 (0)	1 (3.2)	1 (1.6)	
Cirrhosis	25 (83.3)	26 (84.9)	51 (83.6)	0.787
Child–Pugh Class				0.281
A	24 (80)	21 (67.7)	45 (73.8)	
B	6 (20)	10 (32.3)	16 (26.2)	
C	0 (0)	0 (0)	0 (0)	
BCLC class				0.936
A	5 (16.7)	11 (35.5)	16 (26.2)	
B	22 (73.3)	11 (35.5)	33 (54.1)	
C	3 (10)	9 (29)	12 (19.7)	
MELD-Score				0.311
Mean (RAnge)	9 (6–17)	8 (5–20)	8 (5–20)	
Localization				0.270
Right liver lobe	17 (56.7)	11 (35.5)	28 (45.9)	
Left liver lobe	1 (3.3)	6 (19.4)	7 (11.5)	
Both lobes	12 (40)	14 (45.1)	26 (42.6)	
No. of liver lesions				0.416
1 lesion	9 (30)	15 (48.4)	24 (39.3)	
2 lesions	10 (33.3)	5 (16.1)	15 (24.6)	
3 lesions	5 (16.7)	4 (12.9)	9 (14.8)	
4 lesions	3 (10)	4 (12.9)	7 (11.5)	
≥ 5 lesions	3 (10)	3 (9.7)	6 (4.9)	
Initial tumor diameter (mm)	49.54 ± 35.34	38.66 cm ± 48.30	43.15 ± 42.16	0.415
Intial tumor volume (cm^3^)	55.86 ± 206.03	24.56 ± 302.83	37.38 ± 259.03	0.478
No. of TACE sessions				0.491
1	2 (6.7)	2 (6.5)	4 (6.6)	
2	2 (6.7)	1 (3.2)	3 (4.9)	
3	26 (86.6)	28 (90.3)	54 (88.5)	
AFP (ng/ml)				0.697
Mean (Range)	22.1 (1.5–5900)	18.2 (1.9–58,883)	19.2 (1.5–58,883)	

**Fig. 1:**
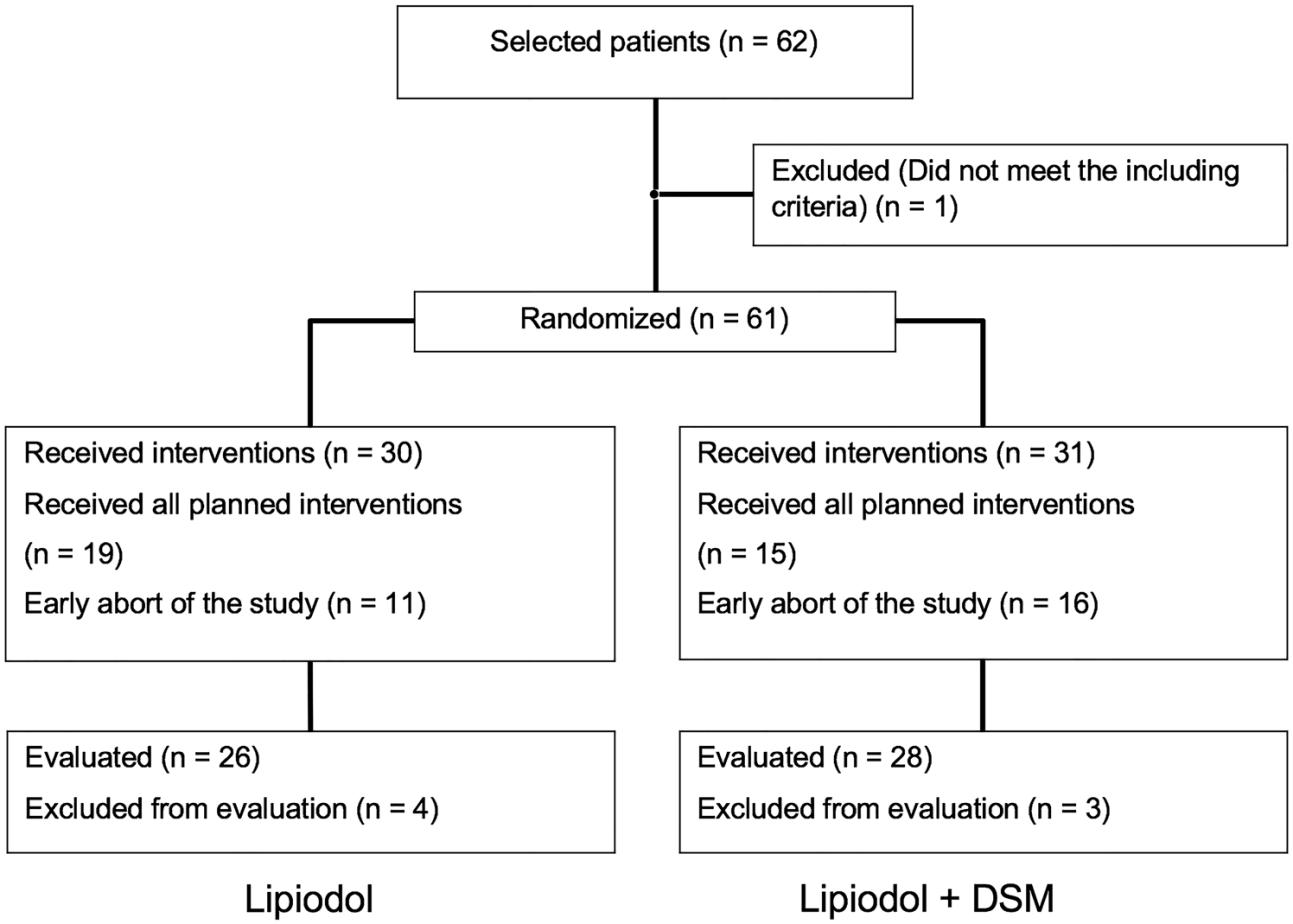
Patient inclusion, exclusion, and randomization diagram

Thirty participants were randomized in the cTACE with Lipiodol-only arm (group 1) and 31 into the cTACE plus DSM arm (group 2). In the Lipiodol-TACE group, 26 participants underwent the study protocol, in 4 patients the therapy was incomplete with 2 cases by decision of the local tumor board for a different treatment regime, in 1 patient due to shift to best supportive care, and also in 1 patient because a withdraw from the study for unspecified reasons. In the cTACE plus DSM group, 28 participants were evaluated. In 3 of the cTACE plus DSM participants the therapy was incomplete, in 2 by decision of the local tumor board for a different treatment regime and in 1 participant with tumor invasion of the portal vein with subsequent stent implantation. Every acquired MRI scan was evaluated for a total of 54 participants (cTACE with Lipiodol-only *n* = 26; cTACE plus DSM *n* = 28) and, ultimately, 7 were excluded due to lack of follow-up imaging (Fig. 1)**.**

### Tumor volume

**Fig. 2:**
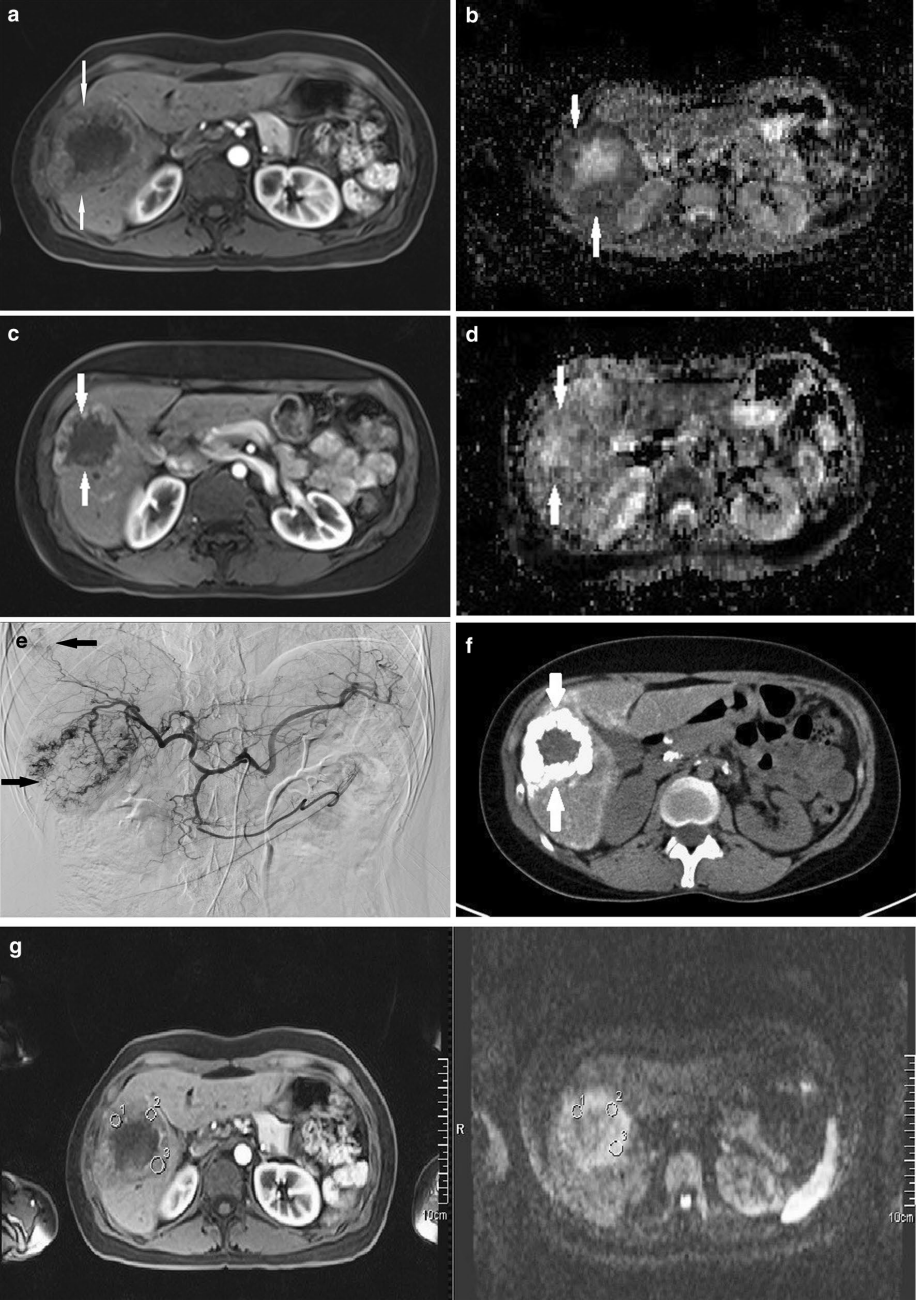
MRI in a representative 40-year-old participant with hepatitis B and cirrhosis who presented with two HCC lesions in segment 7 and segment 8. **a** Axial acquired contrast-enhanced T1-weighted MRI at baseline showing an HCC-typical lesion in segment 7 with central necrosis. **b** Baseline ADC-map demonstrating low ADC-values in the tumor mass and high signal in the necrotic area compared to normal ADC-values. **c** Axial contrast-enhanced T1-weighted MRI performed following the third TACE + DSM showing a RECIST partial response with an increase in size of the necrotic area. **d** ADC-map post-study completion showing increased ADC-values in the tumor mass as a predictor of therapy response. Values in the necrotic area remain increased. **e** Angiography demonstrating typical HCC hypervascularization with additional segment 8 hypervascularity of the second lesion. **f** Computed tomography following a 4-week-interval after the first TACE + DSM demonstrating prolonged Lipiodol uptake in the HCC lesion. **g** A post-contrast T1 sequence was linked to the ADC map for delineation of ROIs on the higher spatial resolution T1 images

For the total study population, a mean reduction of tumor volume measuring 2.76 cm^3^ (± 151.60 cm^3^) was observed (21.45%, ± 62.34%). A greater tumor volume reduction trend was observed for the cTACE plus DSM group (reduction of 3.46 cm^3^, 22.95%) than in the Lipiodol-TACE group (reduction of 1.40 cm^3^, 21.45%) that did not meet statistical significance (*p* = 0.653; *p* = 0.556) (Fig. 2 and Table 3).

**Table 3:** Study results

	Lipiodol (± SD)	Lipiodol + DSM (± SD)	Overall (± SD)	*p*-value
Tumor volume				
Absolute (cm^3^)	− 1.40 (± 88.46)	− 3.46 (± 190.60)	− 2.76 (± 151.60)	0.653
Relative (%)	− 21.45 (± 64.36)	− 22.95 (± 61.39)	− 21.45 (± 62.34)	0.556
Diameter				
Absolute (cm)	− 2.64 (± 15.89)	− 1.63 (± 7.09)	− 1.97 (± 12.08)	0.562
Relative (%)	− 11.86 (± 24.53)	− 4.06 (± 21.01)	− 6.26 (± 22.57)	0.678
mRECIST				**0.010**
Complete response	0	3	3	
Partial response	2	7	9	
Stable disease	21	17	38	
Progressive disease	3	1	4	
Necrotic area				
Absolute (cm)	− 0.88 (± 6.83)	1.10 (± 6.15)	0.98 (± 6.43)	0.222
Relative (%)	− 4.57 (± 42.77)	4.27 (± 38.65)	0.01 (± 40.54)	0.107
Complications				0.103
Grade 1	6	5	11	
Grade 2	1	1	2	
≥ Grade 3	0	0	0	

Bold values indicates significance

*DSM* degradable starch microspheres, *mRECIST* modified response evaluation criteria in solid tumors, *SD* standard deviation

A significant correlation between the initial MRI evaluated tumor volume and the relative volume reduction under therapy was calculated with a correlation coefficient of rho = 0.383 (*p* = 0.004). The initial alpha-fetoprotein **(**AFP) value did not correlate with the volume (*ρ* = 0.131; *p* = 0.890) change following therapy.

### Diameter and mRECIST

Similar to the response evaluation by volume changes under therapy, for the response defined by diameter measurements, no significant differences of the response evaluation by diameter between the control and trial group were seen (*p* = 0.678). Converse to the volume response under therapy, a slight benefit on side of the control group was observed (mean change overall: − 6.26%; using Lipiodol: − 11.86%; or Lipiodol with DSM: − 4.06%), details can be found in Table 3.

Response evaluation by mRECIST revealed significant differences between both groups with benefit for the Lipiodol with DSM group (*p* = 0.010). In detail, comparing the cTACE with Lipiodol-only and cTACE with DSM protocol, complete response (CR) was seen in no case (0%) in group 1 versus 3 cases (10.7%) in group 2, partial response (PR) in 2 cases (7.7%) versus 7 cases (25.0%), stable disease in 21 cases (80.7%) versus 17 cases (60.7%) and a progressive disease in 3 cases (11.5%) versus 1 case (3.6%), respectively (Table 3).

### Necrotic area

A significant correlation between the longest initial diameter of the necrotic area and the diameter of the complete HCC-lesion in the initial MRI (*ρ* = 0.402; *p* = 0.003) and the analogue volume measurement (*ρ* = 0.350; *p* = 0.011) was found. On initial imaging, a total of 28 necrotic lesions were identified in 25 participants. During the course of treatment, 8 new necrotic lesions were identified in 6 of the participants. In 3 patients (2 in the Lipiodol-TACE-group, 1 in the cTACE plus DSM-group) the necrosis resolved under therapy. The mean diameter of necrosis in the initial MRI was 1.27 cm ± 1.80 cm with no difference between the Lipiodol-TACE (0.60 cm ± 1.97 cm) and cTACE plus DSM (0.80 cm ± 2.76 cm) group (*p* = 0.687). During therapy, there was a mean increase of necrosis diameter of 0.98 cm ± 6.43 cm (0.01% ± 40.54%). The necrosis diameter was reduced by 0.88 cm ± 6.83 cm (− 4.47% ± 42.77%) in the cTACE with Lipiodol-only group versus an increase by 1.10 cm ± 6.15 cm (+ 4.27% ± 38.65%) in the cTACE plus DSM group with no significant difference (*p* = 0.222 and *p* = 0.107) (Table 3).

### Complications

In 50 patients, no treatment-related complications were seen. Nine patients presented with a grade 1 complication, post-interventional nausea or fever (14.8%), 6 in group 1 (20.0%) and 5 in group 2 (16.1%). An allergic reaction (grade 2 complication) was seen in 2 patients (3.3%), 1 patient in each group (3.3%; 3.2%). No complications of grade 3 or higher were seen related to the intervention. No significant differences in the occurrence of complications were reported (p = 0.103) (Table 3).

### Survival data

The mean overall survival for the entire cohort was 24.61 months (range 1–54 months). Overall survival for the Lipiodol-TACE group was with 22.77 months (range 2–49 months) shorter than in the cTACE with DSM group (26.32 months, range 1–54 months) with no significant difference (p = 0.844).

**Fig. 3:**
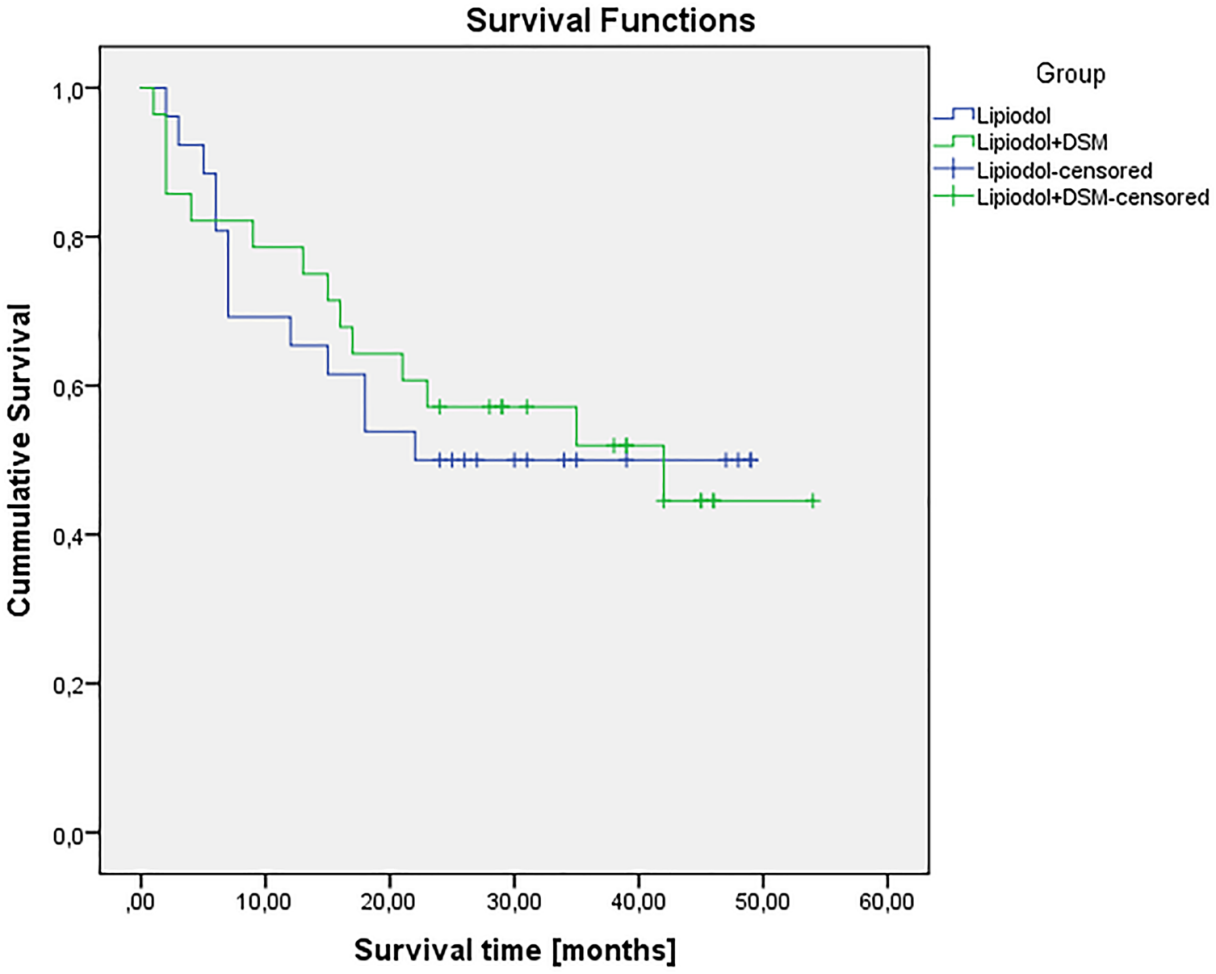
Kaplan– Patient Mayer plot of the study groups

Estimated survival times for the study population are 32.5 months (95% confidence interval: 26.6–38.4), in group 1 29.4 months (95% CI 21.7–37.1) and in group 2 33.4 months (95% CI 25.5–41.4), respectively (Fig. 3).

Using the Cox regression test, longer survival was associated with a low baseline AFP (*p* = 0.002), as well as a smaller lesion volume and diameter (*p* = 0.046 and *p* = 0.005).

Other factors were not associated with the overall survival.

## Discussion

Our study yielded as major finding that we were able to demonstrate a positively potentiating effect of an additional DSM administration compared to cTACE with Lipiodol-only in therapy of HCC by assessment of the tumor response according to mRECIST criteria. No significance was found for therapy response assessed by diameter or tumor volume.

In patients with HCC in a non-resectable situation or requirement for therapeutic bridging, TACE is an established therapy. The major benefit of TACE is the selective or super-selective application of high concentrated chemotherapeutics directly in the tumor feeding vessels minimizing systemic side effects. This feature is supported by the occlusion of these tumor-feeding vessels using a variety of substances with different chemical characteristics. Well established and investigated is the use of Lipiodol for embolization, called the conventional TACE, with a long-term occlusion of the tumor feeding vessels and the tumor area. This was used as standard compared to an additional application on DSM-particles. These particles are used for a transient embolization usually of the edge areas combined with Lipiodol for a long-lasting embolization of the tumor core [15]. An important limitation of all TACE regimes is a high rate of tumor recurrences, especially for large or invasive tumors [16, 17].

However, there is a lack of data regarding the optimal chemotherapeutic and embolization treatment protocol and therapeutic response monitoring strategy. The convenience of serial single-plane tumor diameter measurement has made it the current standard in widely-used response monitoring tools such as mRECIST [14] but there is growing evidence to suggest that volumetric measurement yields consequential differences as expected with aspheric tumor shapes. However, even volumetric measures may be inadequate as Galban et al. have demonstrated that changes in HCC internal tissue architecture can precede volumetric changes [18].

This study revealed a significant benefit for the additional application of DSM combined with Lipiodol compared to Lipiodol-only (cTACE) as an embolization agent according to the tumor response evaluation by mRECIST. No significant benefit but a trend towards the use of DSM was seen in response evaluation by diameter and volume not reaching the level of significance.

It is hypothesized that the additional use of DSM with a transient occlusion of tumor-supplying vasculature decreases the overall VEGF-induced neovascularization following TACE. The underlying concept of this study approach is a long-term embolization of the tumor core using Lipiodol and a temporary embolization of the tumor edge area with DSM is performed for better tumor response. Schicho et al. demonstrated decreased VEGF levels using this combination [19]. With the use of Lipiodol as stand-alone embolic agent and a consecutive long-term embolization of all tumor areas, the core, and the edge, the VEGF response might be even higher and the tumor response limited by early neovascularization. We think, that especially the vital edge of the tumor and not the, often necrotic, core has the crucial impact on the response and limitation of the treatment which might be an explanation for our findings and the benefit of the additional administration of DSM in the tumor-accommodative arteries.

Yamasaki et al. observed a tumor response rate of 80% using Lipiodol with DSM and 40% using Lipiodol only with a significantly longer progression-free interval [20]. Further, there was a lower level of side-effects and increased patient tolerance with TACE plus DSM reported which was not supported by our study [21]. For further studies, the impact of the tumor size on the therapy outcome should be investigated.

Putting the results of this study in the clinical context, for patients requiring TACE as therapy of HCC, using a combination of Lipiodol and DSM as embolization protocol might be favored compared to Lipiodol alone. Experience of the interventional radiologist, local circumstances, and availability should be taken into consideration.

A further approach to categorize the therapy outcome of HCC after TACE is the Liver Imaging Reporting and Data System (LI-RADS) treatment response (LR-TR) algorithm. This algorithm has been proven by Abdel Razek et al. and is established in the clinical routine. As we decided to use the mRECIST system for therapy response evaluation, an additional evaluation based on the LR-TR system might be useful for further studies to investigate the response to thermal ablation or TACE [22, 23].

Limitation of this study includes the limited sample size present in both groups, according to the pilot character of this study, the heterogeneity in participant treatment cycle completion rates, incomplete follow-up evaluations and the comparison limited to two embolization protocols only.

The combination of Lipiodol and degradable starch microspheres (DSM) as agents for embolization of HCC lesions in patients requiring for TACE is favorable compared to Lipiodol alone as standalone embolic agent (cTACE with Lipiodol-only) based in tumor response evaluation by mRECIST. Survival after treatment is not affected by the embolization regime.

## Data Availability

None.

## Declarations

### Clinical trials registration

This study was registered by our local ethical committee. No registration in other trials registries.
